# Understanding and controlling morphology evolution via DIO plasticization in PffBT4T-2OD/PC_71_BM devices

**DOI:** 10.1038/srep44269

**Published:** 2017-03-13

**Authors:** Yiwei Zhang, Andrew J. Parnell, Fabio Pontecchiani, Joshaniel F. K. Cooper, Richard L. Thompson, Richard A. L. Jones, Stephen M. King, David G. Lidzey, Gabriel Bernardo

**Affiliations:** 1 Department of Physics and Astronomy, The University of Sheffield, S3 7RH, UK; 2 ISIS Pulsed Neutron and Muon Source, STFC, Rutherford Appleton Laboratory, Harwell Campus, Oxon, OX11 0QX, UK; 3 Department of Chemistry, Durham University, South Road DH1 3LE, UK

## Abstract

We demonstrate that the inclusion of a small amount of the co-solvent 1,8-diiodooctane in the preparation of a bulk-heterojunction photovoltaic device increases its power conversion efficiency by 20%, through a mechanism of transient plasticisation. We follow the removal of 1,8-diiodooctane directly after spin-coating using ellipsometry and ion beam analysis, while using small angle neutron scattering to characterise the morphological nanostructure evolution of the film. In PffBT4T-2OD/PC_71_BM devices, the power conversion efficiency increases from 7.2% to above 8.7% as a result of the coarsening of the phase domains. This coarsening process is assisted by thermal annealing and the slow evaporation of 1,8-diiodooctane, which we suggest, acts as a plasticiser to promote molecular mobility. Our results show that 1,8-diiodooctane can be completely removed from the film by a thermal annealing process at temperatures ≤100 °C and that there is an interplay between the evaporation rate of 1,8-diiodooctane and the rate of domain coarsening in the plasticized film which helps elucidate the mechanism by which additives improve device efficiency.

Organic photovoltaic solar cells (OPVs) represent a promising approach to generate renewable energy^1^,^2^,^3^. Compared to silicon-based solar cells, OPVs can be processed via solution over much larger areas and using flexible substrates, suggesting possible reductions in module fabrication cost together with the potential to reduce the energy payback time^3^,^4^,^5^,^6^.

In the last decade, bulk heterojunction (BHJ) OPVs based on low band gap copolymers as electron donor and fullerene derivatives as the electron acceptor have developed rapidly, attaining power conversion efficiencies (PCEs) >10% for single layer devices^7^,^8^. The photovoltaic effect in a BHJ commences with the generation of an exciton resulting from the absorption of a photon. Such excitons must be rapidly dissociated at a donor-acceptor interface to avoid recombination, with the charges generated being extracted through a bicontinuous and interpenetrating network of phase-separated fullerene and polymer-rich domains. The typical diffusion length of a singlet-exciton in conjugated polymers is as low as ca. 10 nm^9^,^10^; a length-scale that necessarily defines the size of phase-separation for optimal device efficiency.

Different processing methodologies to optimize the morphology of BHJ films and increase device performance (including the use of thermal annealing^11^,^12^,^13^,^14^,^15^ and the use of solvent additives^16^,^17^,^18^,^19^) are now widely established in the OPV field. Thermal annealing (TA) was the first of these strategies to be explored as it proved most effective when used with crystalline polymers such as P3HT. For example annealing P3HT:PCBM blends at around 150 °C was used to improve the degree of crystallinity of the polymer and thus enhance device efficiency^11^,^12^,^13^,^14^,^15^. This is in contrast with less crystalline or amorphous polymers such as PCDTBT^20^,^21^ in which annealing at temperatures <100 °C has a limited benefit on device PCE mainly through removal of residual solvent rather than by causing a change in film nanostructure^20^, with annealing at higher temperatures causing a drastic decrease in PCE^21^. The use of solvent additives^16^,^17^,^19^ such as 1,8-diiodooctane (DIO) is an alternative strategy which proved to be the most effective with polymers such as PTB7^22^,^23^,^24^ and PBDTTT-EFT^25^. Devices simultaneously treated using both processes (additive and annealing) have also been reported to show significant improved performance compared with those treated with either process alone^26^.

It is now well known that small molecule additives can promote phase segregation^18^,^27^,^28^, as well as polymer crystallization in the BHJ^29^. For example, Chen *et al*.^27^, using AFM, estimated that in the P3HT:PCBM system the addition of 3% v/v DIO to the solution caused an increase in domain sizes in the corresponding BHJ from ~10 nm to ~15 nm. However the mechanism by which this process occurs as well as the early stages of the film drying and nanostructure evolution are still poorly understood.

Among a variety of electron donor materials, poly[(5,6-difluoro-2,1,3-benzothiadiazol-4,7-diyl)-alt-(3,3′″-di(2-octyldodecyl)2,2′;5′,2″;5″,2′″-quaterthiophen-5,5′″-diyl)] (PffBT4T-2OD) has recently attracted attention due to its potential to fabricate high performance OPV devices^8^,^30^. This potential stems from the fact that PffBT4T-2OD exhibits high crystallinity resulting in high hole-mobility, together with the formation of relatively purer polymer domains that allow it to perform well even when used in relatively thick layers (~300 nm) in an OPV device.

Liu *et al*.^8^ have shown that in solution, PffBT4T-2OD exhibits a peculiarly strong temperature-dependent aggregation behavior, characterized by the formation of a gel at room temperature. Consequently, PffBT4T-2OD thin films are always cast from warm solutions (>60 °C), which then aggregate or crystallize during cooling and film formation. Indeed, it has been shown that this aggregation behavior is insensitive to the presence of the fullerene acceptor and can be efficiently used to control the morphology of the corresponding BHJs. This has permitted a near-ideal polymer:fullerene morphology to be created (containing highly crystalline, preferentially orientated, yet small polymer domains) by control over polymer aggregation during solution casting.

Ma *et al*.^30^ have studied the influence of processing parameters such as solution temperature, concentration, spin-rate and solvent quality, as well as the influence of polymer molecular weight on the morphology and properties of PffBT4T-2OD:PC_71_BM solar cells. Here, it was found that the molecular orientation and molecular packing can be tuned by adjusting the spin rate during spin-coating and the solution temperature for blend films, with a low spinning rate and low solution temperature inducing highly ordered face-on polymer packing, and a high spinning rate and high solution temperature producing poorly ordered edge-on polymer packing. The best OPV performance was achieved using films spun-cast at 800 rpm from a solution at 100 °C, creating a smooth film containing sufficient aggregates to yield a favourable morphology.

A detailed study of the processing methodologies to optimize the morphology of PffBT4T-2OD based OPV devices has however not yet been reported in the literature.

Experimentally, many different techniques have been used to study the morphology of polymer:fullerene BHJ systems, however difficulties remain in precisely determining the composition and size of the blend phases, the structure of the interfaces between phases and film morphology as a result of limited contrast between the materials in the blend. Despite the fact that it is possible to distinguish between semi-crystalline conjugated polymers and fullerenes using electron and x-ray based techniques, difficulties remain in determining the structure associated with amorphous materials, and as such it is difficult to probe amorphous regions in conjugated polymer-fullerene blends. Furthermore, techniques such as AFM only allow the morphology of the solid-air interface to be determined, however film structure at this interface is often very different from that in the underlying bulk. One technique that can be used to probe such systems is neutron scattering. Neutron scattering has been highly effective in probing the structure of polymer:fullerene BHJs as contrast between these materials originates from differences in the scattering length density (SLD) of fullerenes compared to protonated conjugated polymers, with no additional deuteration of the components being necessary. For this reason, neutron scattering techniques such as Small Angle Neutron Scattering (SANS)^31^,^32^,^33^,^34^,^35^,^36^ and Neutron Reflectivity^36^,^37^,^38^,^39^,^40^,^41^ have provided new insight into the nanomorphology and vertical layer structure of bulk heterojunctions^42^.

In this paper, we elucidate the mechanism by which 1,8-diiodooctane (DIO) improves the device efficiency of PffBT4T-2OD/PC_71_BM based devices. Through a combination of small angle neutron scattering, ion-beam analysis and ellipsometry we demonstrate that DIO acts as a plasticizer to increase molecular mobility and domain coarsening in a process that is assisted by thermal annealing and in which thermal annealing plays two antagonistic roles; it increases molecular mobility due to increased thermal energy, however it simultaneously promotes the evaporation of the plasticizer (DIO) which consequently decreases molecular mobility. Therefore, the ideal morphology results from a fine balance between these two effects; the evaporation rate of DIO and the rate at which domains coarsen in the plasticized film. We believe that this understanding of the mechanism by which additives improve device efficiency can be used in the development of smart strategies for additive development.

## Results and Discussions

### Photovoltaic performances

**Figure 1:**
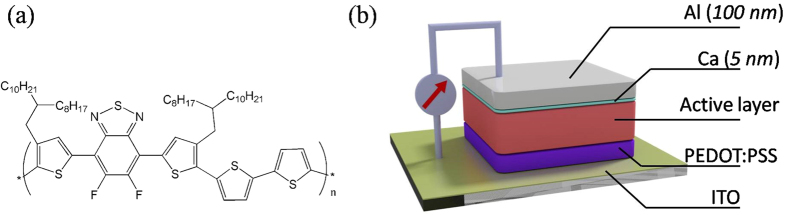
(**a**) Molecular structure of PffBT4T-2OD (**b**) A schematic of the device structure explored.

Figure 1(a) shows the chemical structure of the polymer PffBT4T-2OD. Figure 1(b) shows the structure of the OPV devices studied in this work, comprising ITO/PEDOT:PSS/Active layer/Ca/Al.

**Figure 2:**
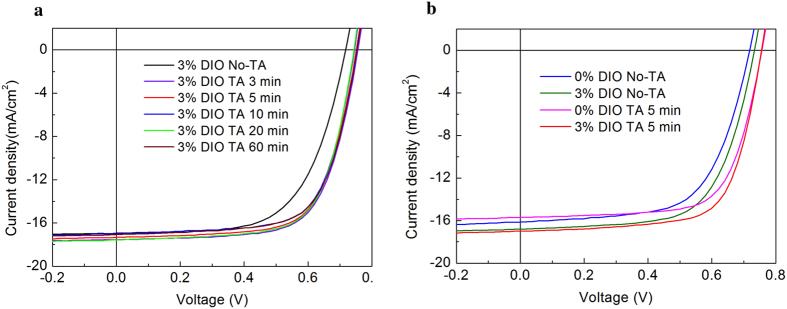
JV curves of devices: (**a**) processed with DIO and with/without different annealing time at 100 °C before cathode evaporation; (**b**) processed without DIO, unannealed and with 5 minutes annealing (before cathode evaporation), and corresponding devices processed with DIO.

Figure 2(a) shows current density-voltage (JV) curves of devices processed with DIO and then annealed for different times as well as an unannealed control. It can be seen that all annealed devices have a higher open-circuit voltage (*V*_*oc*_) and improved fill factor (*FF*) compared to the control. Importantly, these improvements in device metrics all occur in the initial 3 to 5 minutes of annealing, with annealing beyond this time not apparently leading to further improvements in device performance. In fact, we find that longer annealing times lead to a slight decrease in PCE. Figure 2(b) shows representative JV curves of two devices processed without DIO, one unannealed and the other annealed for 5 minutes, together with the JV curves of the corresponding devices processed with DIO as reproduced from Fig. 2(a). As it can be seen, annealed devices (with and without DIO) all have very similar V_oc_ (~0.75 ± 0.01); a value higher than the V_oc_ of the corresponding unannealed devices (~0.72). Furthermore, devices processed with DIO have a larger short-circuit current density (*J*_*sc*_) than the corresponding devices without the DIO. Therefore, while thermal annealing mainly contributes to increase *V*_*oc*_ values, the main effect of DIO is to increase the *J*_*sc*_ values. These effects of thermal annealing on increasing *V*_*oc*_ values and of DIO on increasing *J*_*sc*_ values^18^,^43^ are in agreement with results obtained in other related OPV systems.

The results of Fig. 2(a) and (b) are all summarized in Table 1. Also included in Table 1 are the results obtained for a device processed without DIO and annealed for 10 minutes. It can be seen that in devices without the DIO no further improvements in PCE can be obtained by annealing for times longer than 5 minutes.

The open-circuit voltage (*V*_*oc*_), short-circuit current density (*J*_*sc*_) and the fill-factor (*FF*) in OPV devices are closely correlated to the nano-morphology and the hierarchical structures of the BHJ^44^,^45^,^46^,^47^,^48^,^49^. In order to identify the origin of their enhancement, we have conducted a detailed morphological study using a number of techniques as detailed below.

### Monitoring the removal of DIO

**Figure 3:**
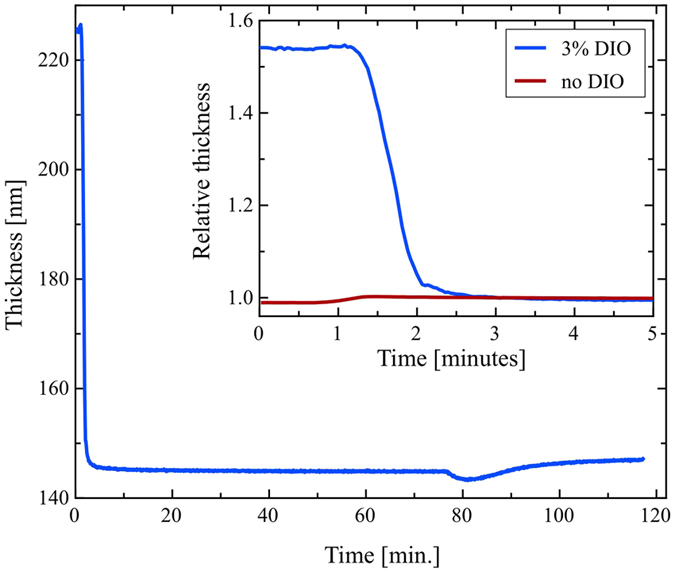
Dynamic data for the isothermal annealing of a PffBT4T-2OD:PC_71_BM blend film annealed at 100 °C. The heating stage was at 100 °C at time t = 1 minute. The inset shows a zoomed in region at the beginning showing the dramatic drop in thickness within the first minute of being at temperature.

Figure 3 shows the variation in thickness of a BHJ film processed from a CB/DCB solvent mixture containing 3 wt% DIO measured using spectroscopic ellipsometry, as a function of time as it is annealed at 100 °C. The inset shows the same data, plotted around the initial 5 minutes for films with and without DIO, with thickness normalised to its value after 5 minutes annealing at 100 °C. The results clearly show that in the case of the film processed with DIO, there is a drop of >30% in film thickness during the first 2 minutes of the annealing; a process that we attribute to the loss of DIO through evaporation. In contrast, the film that does not contain DIO undergoes a small increase in thickness, which we attribute to thermal expansion of the film. This indicates that in this film, the CB/DCB casting solvent underwent complete evaporation during spin-coating. Although CB and DCB have boiling points of 131 °C and 180 °C respectively, this complete evaporation results from the fact that films were spun-cast from a hot solution (110 °C) onto substrates that were held at the same temperature. This result is consistent with other studies on P3HT/PCBM BHJ films^50^ spun-cast from CB or from 1,2,5-trichlorobenzene (b.p. = 214 °C) that dry almost completely in a time period shorter than 10 s if heated to 50 °C. We note however that it is possible that a residual fraction of the casting solvent also evaporates during ellipsometry measurement itself, as measurements are only taken after the sample stage temperature was ramped at 130 °C/min from RT to 100 °C.

We briefly comment on the large thickness change observed in films containing DIO on thermal annealing. Even though DIO is added at low concentration (3% v/v), it is a liquid with extremely low volatility and thus significant quantities are expected to remain in the film after casting. Indeed, the mass of DIO present is nearly three times the total mass of polymer and fullerene. For this reason, the large reduction in thickness observed upon annealing is not surprising.

In Supplementary Figure 1 we show that the annealed BHJ film processed without DIO, shown in the inset of Fig. 3, undergoes a slight contraction (~1%) as it is cooled from 100 °C to 30 °C. We note that the T_g_ of the polymer is close to 100 °C, and thus we attribute this contraction to a relaxation and rearrangement of the polymer molecules. In Supplementary Figure 2 we show the thickness variation of a BHJ film containing DIO, as a function of time under ambient conditions. In this case the DIO evaporation and consequent decrease in film thickness extends over a period of >10 hours, i.e. the drying time is two orders of magnitude longer. In Supplementary Figure 3 we show the thickness profile of two films (both with DIO and without DIO) as they are first heated at 2 °C.min^−1^ from 30 °C to 150 °C. This indicates that when slow heating ramps are applied to such films, the DIO evaporates completely before the temperature reaches 80 °C. In Supplementary Figure 4 we plot the thickness of each film on cooling and thus determine T_g_ from a change in the thermal expansion coefficient of the polymer using previously described methods^20^,^51^. The T_g_ as determined by this method is ~92 °C. In Supplementary Figure 5 we plot the differential scanning calorimetry curve for the pure PffBT4T-2OD polymer showing an absence of any crystallization peaks in the temperature range 25–150 °C. This excludes the possibility of any low-temperature re-crystallization processes that could influence the morphology of the BHJ.

**Figure 4:**
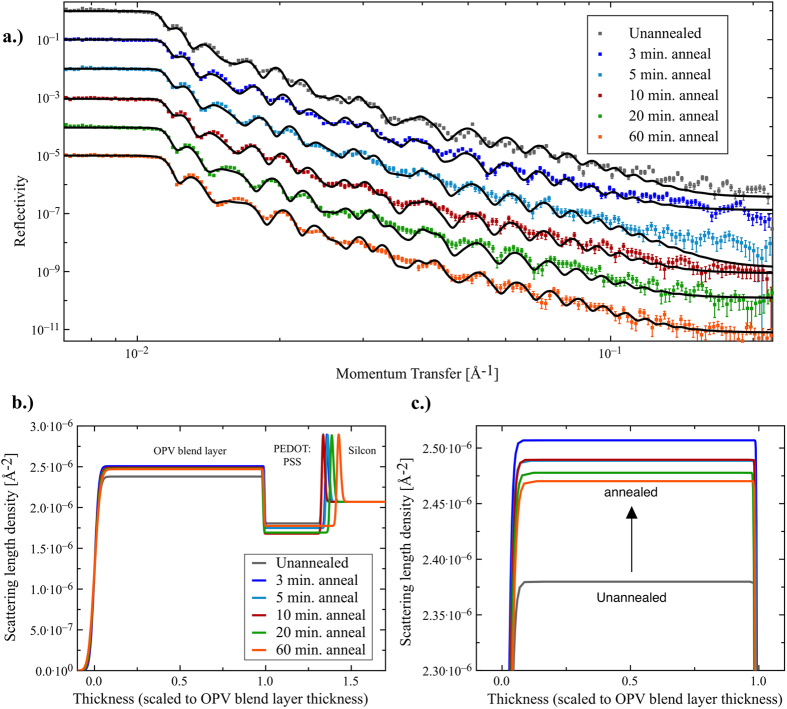
(**a**) Fitted neutron reflectivity data for a series of PffBT4T-2OD:PC_71_BM blend films from unannealed through annealing conditions at 100 °C for 3, 5, 10, 20 and 60 minutes; the data is offset by a decade to aid comparison (**b**) The scattering length density (SLD) profiles generated from the NR fitted data. The thickness is scaled to the thickness of the OPV blend layer to aid comparisons in the profiles; (**c**) a zoomed in view of the change in scattering length density from the unannealed sample, showing the change in SLD upwards after thermal annealing.

In Figure 4(a) we plot neutron reflectivity spectra for the OPV blend films processed with DIO for different annealing times as shown. Figure 4(b) plots scattering length density plots normal to the film surface determined from the reflectivity spectra. The different layers (silicon, PEDOT:PSS and the active layer blend) are readily identifiable due to their different scattering length densities, allowing us to determine the changes in composition within the blend layer itself. Assuming the densities for PffBT4T-2OD and PC_71_BM to be 1.0 g.cm^−3^ and 1.5 g.cm^−3^ respectively^31^, then a 1:1.2 mass ratio of PffBT4T-2OD:PC_71_BM corresponds to volume percentages of PffBT4T-2OD and PC_71_BM of 55.6% and 44.4% respectively. Using the SasView software^52^, we calculate scattering length densities for the PffBT4T-2OD and PC_71_BM to be 0.72 × 10^−6^ Å^−2^ and 4.42 × 10^−6^ Å^−2^ respectively, indicating a SLD value for the active layer of 2.36 × 10^−6^ Å^−2^. The calculated SLD values for the solvents used are as follows: dichlorobenzene = 2.36 × 10^−6^ Å^−2^ (close to the average BHJ SLD value); chlorobenzene = 1.83 × 10^−6^ Å^−2^ and 1,8-diiodooctane (DIO) = 0.118 × 10^−6^ Å^−2^.

By fitting the NR data, we obtain effective SLD of the unannealed blend layer and blend layer after 3 minutes annealing of 2.38 × 10^−6^ Å^−2^ and 2.50 × 10^−6^ Å^−2^ respectively. This increase in the SLD of the BHJ after 3 minutes annealing most likely results from the loss of DIO via evaporation. After longer annealing times we determine a slight drop in the SLD of the BHJ; a result we speculate might originate from aggregation of PC_71_BM occurring at the film surface. However, we cannot exclude difference in SLD resulting from sample-to-sample variability.

We have used ion-beam analysis (Rutherford backscattering, RBS) to explore the evaporation of DIO from the films. RBS experiments are highly sensitive to the presence of DIO as the iodine nuclei are by far the heaviest atoms in the sample and thus ion-recoils from iodine are detected at significantly higher energy than recoils from other nuclei within the film or substrate. Ion-beam experiments do not require the time-consuming sample alignment and heating ramp steps involved in ellipsometry and thus can more accurately follow the kinetics of evaporation in the earliest stages of film drying.

Typical ion-beam data and fits are given in Supplementary Information, Supplementary Figures 6 and 7. Here, data is presented for spun-cast samples stored under different experimental conditions (samples annealed at 100 °C and 1 atmosphere, samples placed inside a vacuum chamber at ~0.01 atm and room temperature and samples kept at 1 atm and room temperature). The fits were based on a model composition profile (Equation 1) in which the DIO concentration, *ϕ*_*DIO*_, was assumed to be largely independent of depth, except for a diffuse region towards the buried interface.


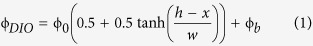


Here, *ϕ*_*0*_ is the maximum concentration of DIO at the film surface, *h* denotes the thickness of the DIO rich layer, *w* was the width of the interface, or variation in film thickness and *ϕ*_*b*_ corresponds to the DIO concentration at greater depths below the film surface.

The data at recoil energy 1255 keV and above can only correspond to recoils from DIO in the sample, which is the only source of sufficiently high mass nuclei to give rise to such high energy recoils. In the case of annealed samples the signal decreases systematically in height and width with increasing annealing time, corresponding to a loss of DIO from the sample with annealing. For every film except the unannealed sample, *ϕ*_*b*_ was found to be zero, and for this case it is likely that significant thickness variations in the freshly prepared film surface are responsible for this result. Nevertheless the very large signal at higher energy when compared to the signal at lower energy from the substrate is consistent with a high concentration of DIO initially present. Interestingly, the fit to the 1 min sample includes a significant tail in the low energy part of the RBS data (~1255 keV) corresponding to a diffuse composition profile for DIO. This feature of the data coupled with the sharp edges at 1065 keV (sulfur surface) and 1616 keV (iodine surface) indicates that at very early stages of annealing, DIO may diffuse into the lower PEDOT layer of the sample before eventually evaporating at longer times. This finding is consistent with other work by Yi *et al*.^53^ who report that solvent additives may affect the PEDOT:PSS layer. At longer annealing times, 3, 5 and 20 minutes, the fits and peaks due to DIO and the sulfur rich layers are consistent, and confirm that the DIO maintains an even vertical distribution within the film as it evaporates.

Our ion-beam analysis measurements shown in Supplementary Figure 7 suggest that thermal annealing the BHJ films at 100 °C is enough to reduce the DIO concentration through evaporation by nearly three decades in around 20 minutes, even though this temperature is well below the DIO boiling temperature (167–169 °C at a base pressure of 6 mmHg). We note similar work by de Villers *et al*.^54^, in which an annealing temperature of 175 °C was used to facilitate the removal of DIO from a BHJ film in order to reduce photo-degradation. We also find that the removal of DIO at room temperature using a vacuum (~0.01 atm) proceeds much more slowly than occurs via thermal annealing (see Supplementary Figure 7). Interestingly, we find that there is still a substantial amount of DIO remaining in the sample after ~24 hours under ambient temperature and pressure.

### Film morphology

We have used scanning force microscopy (SFM) to study the surface morphology of films processed with and without DIO, both before and after annealing and later dried under Ultra-High-Vacuum (UHV). We present a selection of images in Supplementary Figure 8. Unfortunately the interpretation of these figures is ambiguous. We also note that SFM only probes the surface morphology of the film that may be very different from that of the underlying bulk material.

**Figure 5:**
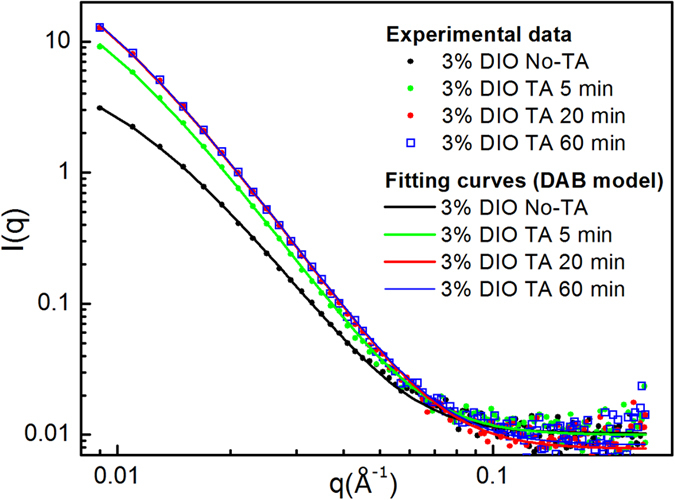
SANS intensity (I) as a function of scattering vector (q) for the non-annealed film and the film annealed for 5, 20 and 60 minutes.

In order to probe the bulk morphology of the film, we have used SANS. Whilst SANS has been previously used to study OPV blends, in most instances this technique has only been applied to thick solvent cast films^33^,^34^ rather than thin films characteristic of those used in devices. Work by Jones *et al*.^55^,^56^ showed that it was possible to measure a SANS signal from a stack of multiple thin film samples. We have used this method, and in Fig. 5 we show SANS spectra recorded from a stack of 12 PffBT4T-2OD/PC_71_BM films on quartz discs (equivalent to a total dry active layer thickness of ~2.4 μm), with intensity plotted versus scattering vector, for both the annealed and unannealed samples. In SANS, the intensity is proportional to the number, size and contrast of the scattering entities in a sample, while the *q*-dependence of the intensity is related to their shape and local arrangement. As can be seen, there is a clear and gradual increase in scattering intensity at small *q* values after annealing for 5 and 20 minutes. However, samples annealed for 20 and 60 minutes show very similar scattering intensity, suggesting that after 20 minutes the system has evolved to a situation of nearly thermodynamic equilibrium.

A simple power law fit to the *q* data in the linear region between 0.01 and 0.07 indicates that the intensity decays with a power law relationship proportional to *q*^−*α*^ with α being between 2.9 and 3.5 (see details in Table 2). Such exponents are consistent with moderately-segregated network like structures. The full data range was then fitted using the Debye-Bueche (DB) or Debye-Anderson-Brumberger (DAB) model^57^,^58^ (Equation 2). This model calculates the scattering from a randomly distributed, two-phase system that is characterized by a single length scale - the correlation length, L - that is a measure of the average spacing between regions of two different phases (1 and 2). This function has the form


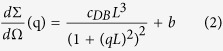


where the scaling factor *C*_*DB*_ = *8π(Δρ*)^*2*^*ϕ*_*1*_*ϕ*_*2*_ and *Δρ* is the neutron scattering length density difference between the phases having volume fractions of *ϕ*_*1*_ and *ϕ*_*2*_. The second term on the right hand side of the equation (*b*) is the background intensity that includes both instrumental and sample specific factors, i.e. the incoherent scattering intensity. As shown in Fig. 5, the DB model gives a good description of all the data. The values obtained from the fitting for *C*_*DB*_ and *L* using equation 2 are given in Table 2. Also shown are the corresponding values of the normalized χ^2^ that confirm the good quality of the fits. For completeness, Table 2 also includes fitting parameters for films that were also processed with and without DIO both before and after 5 minutes annealing at 100 °C.

We have also fitted the full data range using a conceptually different model, namely the Mass Fractal model^59^. This calculates the scattering from fractal-like aggregates consisting of spherical primary particles of radius *R*, with *R* assumed to be 5 Å (the approximate radius of a PC_71_BM molecule). Here, we have obtained very similar characteristic length scales as those obtained using the DAB model (see Supplementary Figure 9 and Supplementary Table 1).

To understand our SANS data, we assume that the morphology of the BHJ is an irregular two-phase system, as illustrated in Supplementary Figure 10. For this reason it is difficult or even misleading to define an ‘average domain size’. Instead we define two transversal chord lengths L_1_ and L_2_ as being the distance that a line (chord), crossing the system in any arbitrary direction, travels inside domains of phase 1 or of phase 2. Each chord length will of course have a distribution of values, but its average value, or ‘Porod-length’, can be calculated by dividing the (three-dimensional) value of the density correlation length by the volume fraction of the *opposite* phase (generally assumed to be equal to the volume fraction of the relevant component)^32^,^60^. Assuming, as an approximation, that the volume fractions of polymer and fullerene in the BHJ are the same (i.e., Φ_1_ = Φ_2_ = 0.5), then we arrive at L_1_ and L_2_ values ~18 nm before annealing and ~26 nm after 5 minutes annealing. We note however that as we make several assumptions in this calculation, these values should be considered only as first-order estimates.

We have also characterised the UV-Vis absorption of PffBT4T-2OD:PC_71_BM blend films processed with DIO before and after annealing, as shown in Supplementary Figure 11 where all spectra have been normalized based on the intensity of the 0–1 transition peak at ~640 nm. It can be seen that the region at ~700 nm corresponding to the 0–0 transition peak remains almost unchanged with thermal annealing which suggests that no significant change in crystallinity has occurred^8^. In the same Figure we show as inset the EQE spectra for two devices processed with DIO, with one BHJ being subjected to 5 minutes of thermal annealing at 100 °C and the other BHJ remaining unannealed. As shown, the EQE of each device are similar to the absorption spectra of the BHJ as expected^8^. We find that annealed device has a slightly higher peak EQE at 500 nm compared to the unannealed device (75% compared to 70%), however over most of the absorption bandwidth the measured EQE spectra are similar. There is a small increase in the integrated EQE (3.8%) with annealing, this result is consistent with the fact that thermal annealing for 5 minutes does not result in a significant increase in J_sc_ for devices that contain DIO (see Table 1).

The effective separation and transport of the photo-generated charge carriers is crucial to device performance and in order to investigate the yield of exciton generation, we measured the photoluminescence (PL) spectrum from the different films as shown in Supplementary Figure 12. From 0 to 3 minutes annealing, there is a dramatic increase in PL intensity that then remains almost unchanged over a following 3 to 60 minutes annealing time. This large increase in PL intensity, which is associated with a corresponding decrease in exciton quenching, suggests that upon annealing the films containing DIO the phase domains become purer which is also in agreement with our SANS observations.

We have used grazing incidence wide-angle X-ray scattering (GIWAXS) to study the impact of the different processing conditions on the crystalline structure of the system, and the results are shown in Supplementary Figure 13 and 14. The dominant orientation for the blends without DIO is a face-on lamellar structure. Annealing this blend for 5 minutes improves the out-of-plane ordering, and we can see well-defined peaks for the face-on lamellar packing at 0.29 Å^−1^ along with higher orders at 0.59 Å^−1^ and 0.88 Å^−1^. The lamellar stacking spacing for all the samples studied is somewhat greater than that seen for pure PffBT4T-4OD (q = 0.28 Å^−1^)^30^ with values closer to ~0.3 Å^−1^, which means a smaller spacing. The same is true for the π-π stacking distance, with values ~1.77 Å^−1^ compared to 1.74 Å^−1^ for the pure polymer^30^. We also observe a broad halo at q = 1.4 Å^−1^ characteristic of PC_71_BM aggregation. In terms of the sample with 3% DIO, here we see much more isotropic orientation of the polymer crystallite orientation, with Debye-Scherrer like rings instead of the strong out-of-plane orientation dominance seen without DIO, showing that the crystals exist in all possible orientation angles relative to the substrate surface. We think that this is due to the difference in drying dynamics, insofar as the films without DIO are dry and vitrified after spin coating has finished. Whereas the films with DIO are still solvated by DIO and therefore mobile, the strong shear gradient from spin coating has been removed before the blend layer has vitrified. These results are in agreement with our SANS results in showing that DIO facilitates changes in the morphology of the film.

**Figure 6:**
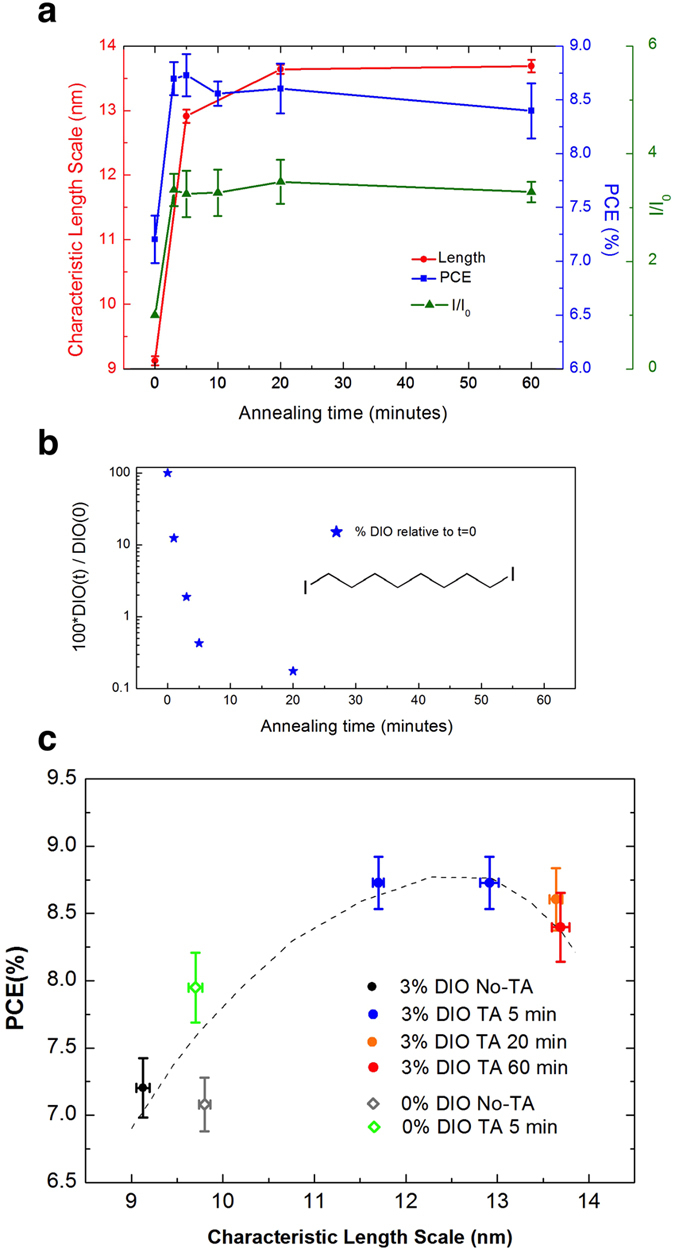
(**a**) Correlation between power conversion efficiency (PCE), characteristic length scale and PL intensity as a function of the annealing time for devices processed using DIO; (**b**) Removal of DIO as a function of time at 100 °C; (**c**) Correlation between PCE and characteristic length scale for samples processed in different ways, i.e. with/without DIO and with/without annealing.

To compare our findings determined from the different characterisation techniques, we plotted in Fig. 6(a) the characteristic length scale (correlation length), PCE and the PL intensity ratios (recorded at 740 nm) of annealed films (I) and un-annealed film (I_0_), as a function of annealing time. For the sake of comparison, Fig. 6(b) plots the relative fraction of DIO in the films as a function of time during annealing at 100 °C as measured using ion-beam analysis. In Fig. 6(c) we plot device PCE as a function of the characteristic length scale determined from SANS, where we have aggregated data from all processing conditions (namely with DIO before annealing and after annealing at 100 °C for times of 5 min, 20 min and 60 min, without DIO and without annealing and without DIO and with 5 minutes of annealing at 100 °C).

Comparing data, it can be seen in Fig. 6(a) that significant domain coarsening in films processed with DIO occurs within the first 5 minutes of annealing. This coarsening then slows progressively, with no further domain coarsening observed for annealing times greater than approximately 20 minutes. As can be seen in Fig. 6(b) this time-scale seems correlated with the time required to evaporate the DIO upon annealing. This correlation of time-scales suggests that DIO acts as a plasticizer that increases the molecular mobility, and that phase coarsening only occurs while the residual DIO is present. It can also be seen that annealing results in a large increase in PL emission intensity. This observation is also consistent with increased phase separation of the polymer and PC_71_BM (as a result of domain coarsening) and thus reduced exciton quenching via charge-separation. From our aggregated data presented in Fig. 6(c), it can be seen that the highest PCE (obtained after 5 minutes annealing) is associated with a characteristic length scale in the range 11.7–12.9 nm. Furthermore, we find that coarsening of the blend morphology beyond this length scale is associated with a slight decrease in PV efficiency. We note that the characteristic length scales necessary to produce the highest device PCEs can only be attained through a simultaneous combination of the use of a DIO additive and appropriate thermal annealing.

## Conclusions

In conclusion therefore, we have explored the morphology of a PffBT4T-2OD/PC_71_BM PV applicable blend film during thermal annealing using a variety of optical and structural probes. Neutron reflectivity measurements indicate that the vertical distribution of PC_71_BM within such films is fairly homogeneous, a feature that is qualitatively different from other polymer:fullerene blends^37^,^38^,^39^,^40^. Importantly, we find that DIO improves the PCE of PffBT4T-2OD/PC_71_BM OPV devices upon annealing by plasticizing the film. This plasticization enables the coarsening of the nanomorphology of the BHJ during annealing at 100 °C, with characteristic length scales having an initial value of 9–10 nm reaching a value of 12–13 nm after around 5 minutes. Although further annealing leads to further small increases in characteristic length scale (to ~14 nm) this is apparently detrimental to device PCE.

Our results strongly suggest therefore that there is an interplay between the kinetics of domain coarsening and the evaporation of DIO. Indeed, the amount of plasticizer DIO present in the film drops abruptly during annealing due to evaporation, being almost completely removed from the film after 20 minutes. On removal of the DIO, the rate of domain coarsening drops abruptly, with the final film phase morphology becoming effectively locked-in. We believe therefore that the domain size within a polymer:fullerene blend may be controllable through the selection of plasticizers having different evaporation rates, with our results providing a rational framework for the development of improved processing routes for PV-applicable BHJ materials.

## Experimental Section

### Materials

PEDOT:PSS (HC Stark CleviosAI4083) and PC_71_BM were purchased from Ossila Ltd. The polymer PffBT4T-2OD has the chemical structure shown in Fig. 1(a), Mn = 54,900 g.mol^−1^ and Mw = 117,800 g.mol^−1^ and was purchased from California Organic Semiconductor Inc. The solvents used were purchased from Sigma-Aldrich (chlorobenzene (CB), o-dichlorobenzene (DCB) and 1,8-diiodooctane (DIO)) and were all high purity grade.

### Device fabrication and measurement

The OPV devices studied in this work had the standard structure ITO/HTL/Active layer/Ca/Al as shown in Fig. 1(b). Poly(3,4-ethylenedioxy-thiophene): poly(styrene sulfonic acid) (PEDOT:PSS) was used as the hole transport layer. The active layer was cast from a solution of chlorobenzene and o-dichlorobenzene (1:1 volume ratio) with 3% DIO (volume percentage) as a solvent additive, with the polymer and PC_71_BM having a relative concentration of 9 mg/ml and 10.8 mg/ml respectively. The active layers were spin coated from pre-heated solutions (110 °C) at a spin speed of 1000 rpm onto the PEDOT:PSS/glass substrate that was pre-heated to 110 °C. The active layer was spin-cast in a nitrogen filled glove box, with the films produced having a thickness of ~300 nm as measured with ellipsometry. The films were then left inside the glove box for 3 hours to dry, after which they were placed into a vacuum chamber with a pressure ~1 mbar for another hour, before being annealed at 100 °C for different times (3 minutes, 5 minutes, 10 minutes, 20 minutes and 60 minutes). Some of the films were left unannealed for reference. A cathode composed of 5 nm Calcium (Ca) and 100 nm Aluminium (Al) was then evaporated sequentially on top of the active layer under a vacuum <2 × 10^−6^ mbar to form the top electrode contact. Finally, the devices were encapsulated using UV-cured epoxy (E131, Ossila Ltd) and a glass slide.

Photovoltaic properties of the devices were determined using a Newport 92251A-1000 AM 1.5 solar simulator which was calibrated using an NREL standard silicon solar cell to ensure an irradiance level of 1000 W/m^2^. An aperture mask was utilised to limit the light-exposed area of the device to 2.6 mm^2^.

External quantum efficiency (EQE) measurements were made using a halogen lamp light source in conjunction with a monochromator. The system was calibrated using a Newport 818-UV calibrated silicon photodiode to measure a reference spectrum of the light source.

### Spectroscopic ellipsometry characterization

Spectroscopic ellipsometry (M2000v, J.A. Woollam Co. ellipsometer) was used to determine film thicknesses at room temperature and to measure the drying dynamics of the organic layers, which comprise the (PEDOT:PSS) and the PffBT4T-2OD:PC_71_BM blend layer, over time as they were annealed at 100 °C. We used a Cauchy model to fit Ψ (the ratio of the amplitude of the incident and reflected light beams) and Δ (the ratio of the phase lag of the incident and reflected light beams) over the wavelength range in which the film is optically transparent (840 to 1000 nm). For measurements under annealing conditions, thin films prepared as previously described, were placed on a Linkam heating/cooling stage. The cell had two transparent windows to allow transmission of the polarized incident and reflected ellipsometry beams. The films were rapidly heated from 25 °C to 100 °C and left at 100 °C for 1 hour, with Ψ and Δ recorded as a function of annealing time. This permitted the evolution in thickness to be followed throughout the annealing process.

### Neutron Reflectivity (NR) characterization

For NR characterization, the thin films were spun-cast on polished 4.0 mm thick circular silicon wafers (Prolog Semicor, Ukraine) with diameter 50.0 mm pre-coated with PEDOT:PSS. The neutron reflectivity data was measured at the ISIS neutron spallation source (Oxfordshire, UK) using the INTER instrument, which has a useable incident neutron wavelength range from 1.5 Å–17 Å. Two angles were collected to cover the required momentum transfer range using incident angles of 0.5° and 2.3°. The data was fitted using the ellipsometry measured thicknesses as the starting point and comprising two layers, the bottom being PEDOT:PSS and the top being the OPV blend layer. NR data was modeled using the scheme of Névot and Croce^61^ and the parameter values for the OPV blend layer were allowed to float and converge to a global minimum whilst constant values were used for the PEDOT:PSS layer (thickness, roughness and scattering length density). The fitting was performed using a least-squares fitting approach, all of the fits converged on chi squared per point values of less than 20, thermal annealing times 5 minutes and longer gave chi squared per point value less than 6.

### Ion-Beam Analysis

Thin films were spun-cast on rectangular shaped and polished silicon oxide substrates (S146 from Ossila) of size 20 mm × 15 mm that were pre-coated with PEDOT:PSS. Ion beam analysis (Rutherford backscattering spectroscopy, RBS) was carried out using an NEC 5SDH Pelletron accelerator to deliver a 1.812 MeV ^4^He^+^ beam to the sample surface. The beam was 2.4 mm diameter, and it was established that at least 1 μC ^4^He^+^ ions could be incident at 90° to the sample surface before any artefacts due to beam damage became apparent. The energy of backscattered ions was determined with a 1.5 mm thick PIPS detector at 170° to the incident beam. Recoils from iodine are detected at significantly higher energy than recoils from other nuclei within the film and substrate. Quantification of the residual DIO in spun-cast films was carried out using the Surrey University Datafurnace software (WiNDF 9.3.68 running NDF v9.6a).

### Scanning force microscopy characterization

Scanning Force Microscopy (Dimension 3100, Veeco) (Tapping Mode) was used to image the surface morphology of the PffBT4T-2OD:PC_71_BM BHJ thin films before and after annealing.

### Small Angle Neutron Scattering (SANS) characterization

For SANS characterization, films were spun-cast on 0.5 mm thick Spectrosil^TM^ quartz slides (Knight Optical WHQ 1500-C) (15 mm diameter) that had also been pre-coated with PEDOT:PSS. The active layers were also spin-coated from pre-heated solutions (~110 °C) onto pre-heated substrates (~110 °C) at a spin rate of 1000 rpm. These films were also dried for 3 hours and then annealed at 100 °C for different times. Stacks of 12 films on quartz discs (equivalent to a total active dry layer thickness of ~2.4 μm [12 × 200 nm]), were assembled in order to generate good signal to noise statistics in the SANS signal.

SANS data from the stacks of solid thin films were acquired on the LOQ diffractometer at the ISIS Pulsed Neutron Source (Didcot, UK)^62^. Due to the high carbon to hydrogen content in PC_71_BM, there is a naturally high neutron scattering length density contrast with the hydrogenous PffBT4T-2OD polymer that removes the need for isotopic substitution (deuteration). LOQ is a fixed-geometry “white beam” time-of-flight instrument which utilizes a neutron beam modulated at 25 Hz and having a wavelength range between 2 and 10 Å. Data are simultaneously recorded on two, two-dimensional, position-sensitive neutron detectors, to provide a simultaneous measure of scattering over the q-range 0.008–1.6 Å^−1^ (q = 4π/λ sin θ/2, where θ is the scattering angle). Each sample stack and background sample were typically measured for 1 to 2 hours in order to gather data having sufficiently high statistical precision. The background sample had a PEDOT:PSS layer deposited. Each raw scattering data set was then radially-averaged, corrected for the detector efficiency, sample transmission and background scattering and then converted to scattering cross-section data and plotted on an absolute scale (∂Σ/∂Ω vs q) using Mantid software^63^. For convenience, we shall follow the normal convention of referring to ∂Σ/∂Ω as intensity (*I*). The corrected data were then fitted to appropriate models using SasView software (Version 3.1.1)^52^.

### UV-Vis absorption and photoluminescence emission measurements

UV-Vis absorption spectroscopy was performed using a Horiba Fluoromax-4. Photoluminescence (PL) emission^64^ was generated from PffBT4T-2OD:PC_71_BM thin films (deposited onto a glass slide and annealed using the conditions described above) following excitation using a 532 nm diode pumped solid state laser. Emission was then imaged into a Jobin Yvon Triax 320 spectrometer, with spectra recorded using a liquid nitrogen cooled CCD. A long pass filter with a cut off of 532 nm was placed before the entrance to the spectrometer to reduce scattered laser-light.

### Grazing Incidence Wide-Angle X-ray Scattering (GIWAXS) measurements

GIWAXS measurements were performed on a Xeuss 2.0 SAXS/WAXS laboratory beamline using a liquid Gallium MetalJet (Excillum) x-ray source (9.2 keV, 1.34 Å). The scattered X-rays were detected using a Pilatus3R 1 M detector. Samples were prepared on PEDOT coated Silicon substrates following a procedure identical to that used in the preparation of actual devices.

### Differential Scanning Calorimetry (DSC) measurements

DSC measurements were performed under nitrogen on a Perkin Elmer Pyris-1 calorimeter, where temperature and heat capacity were calibrated against a sapphire standard. Two heating-cooling cycles were run from 25 to 150 °C at 10 °C.min^−1^.

## Additional Information

**How to cite this article**: Zhang, Y. *et al*. Understanding and controlling morphology evolution via DIO plasticization in PffBT4T-2OD/PC_71_BM devices. *Sci. Rep.* **7**, 44269; doi: 10.1038/srep44269 (2017).

**Publisher's note:** Springer Nature remains neutral with regard to jurisdictional claims in published maps and institutional affiliations.

## Supplementary Material

Supplementary Information

## Figures and Tables

**Table 1:** Device metrics showing the peak and (average) values for PCE, V_oc_, FF and J_sc_ for devices with and without DIO and various thermal annealing times.

	Anneal time	PCE (%)	V_oc_ (V)	FF (%)	J_sc_ (mA/cm^2^)
With DIO 3 wt%	No anneal	7.60 (7.20 ± 0.22)	0.72 (0.72 ± 0.007)	62.49 (60.18 ± 1.87)	−16.94 (−16.53 ± 0.59)
	3 minutes	9.02 (8.70 ± 0.15)	0.76 (0.75 ± 0.002)	67.96 (68.37 ± 0.83)	−17.55 (−16.88 ± 0.40)
	5 minutes	8.90 (8.73 ± 0.20)	0.75 (0.75 ± 0.002)	68.34 (68.04 ± 1.53)	−17.33 (−17.09 ± 0.30)
	10 minutes	8.73 (8.56 ± 0.11)	0.74 (0.75 ± 0.004)	68.96 (68.00 ± 0.51)	−16.99 (−16.83 ± 0.19)
	20 minutes	8.95 (8.61 ± 0.23)	0.74 (0.75 ± 0.005)	68.52 (68.13 ± 0.57)	−17.58 (−16.85 ± 0.39)
	60 minutes	8.75 (8.40 ± 0.25)	0.75 (0.75 ± 0.002)	68.06 (66.80 ± 1.28)	−17.07 (−16.73 ± 0.31)
Without DIO	No anneal	7.29 (7.08 ± 0.20)	0.72 (0.72 ± 0.01)	62.93 (62.30 ± 0.56)	−16.13 (−15.83 ± 0.30)
	5 minutes	8.22 (7.95 ± 0.26)	0.76 (0.75 ± 0.01)	69.25 (68.34 ± 0.90)	−15.69 (−15.40 ± 0.30)
	10 minutes	8.25 (7.96 ± 0.21)	0.75 (0.75 ± 0.01)	72.69 (71.06 ± 0.81)	−15.08 (−14.89 ± 0.29)

**Table 2:** 1^st^ column: α values obtained by fitting the experimental data between q = 0.01 and q = 0.07 using the power law model (*q* ^*−α*^); 2^nd^ and 3^rd^ columns: scaling factors (C_DB_) and correlation lengths (L) obtained by fitting the experimental data using the Debye-Anderson-Brumberger (DAB) model in the interval q = 0.009–0.1.

	Sample	α (q^−α^)	Scaling factor C_DB_	Length L (Å)	(χ^2^/Npts)
With DIO 3 wt%	No-anneal	2.87	1.17 × 10^−5^	91.3 ± 0.7	0.72
	5 min anneal	3.37 (3.25)	2.44 × 10^−5^ (2.84 × 10^−6^)	129.1 ± 1.0 (116.6 ± 3.9)	2.03 (0.72)
	20 min anneal	3.46	3.29 × 10^−5^	136.4 ± 0.7	4.62
	60 min anneal	3.47	3.32 × 10^−5^	136.9 ± 1.0	3.26
Without DIO	No-anneal	3.08	1.94 × 10^−5^	98.4 ± 1.2	0.96
	5 min anneal	3.12	1.98 × 10^−5^	97.4 ± 1.1	1.38

The values inside the brackets for the DIO sample with 5 min thermal annealing correspond to a sample repeat prepared under similar conditions.
