# Supplementary material for: Understanding and controlling morphology evolution via DIO plasticization in PffBT4T-2OD/PC71BM devices
Source: Sci Rep. 2017 Mar 13;7:44269. doi: 10.1038/srep44269 (PMC5347161; doi:10.1038/srep44269)
Supplement: Supplementary Information [file srep44269-s1.pdf]

SUPPLEMENTARY INFORMATION

Understanding and controlling morphology evolution via DIO plasticization in PffBT4T-2OD/$PC_{71}BM$ devices

Yiwei Zhang[1], Andrew J. Parnell[1], Fabio Pontecchiani[1], Joshaniel F. K. Cooper[2], Richard L. Thompson[3], Richard A. L. Jones[1], Stephen M. King[2], David G. Lidzey[1,*], Gabriel Bernardo[1,*]

[1]Department of Physics and Astronomy, The University of Sheffield, S3 7RH, UK

[2]ISIS Pulsed Neutron and Muon Source, STFC, Rutherford Appleton Laboratory, Harwell Campus, Oxon, OX11 0QX, UK

[3]Department of Chemistry, Durham University, South Road DH1 3LE, UK

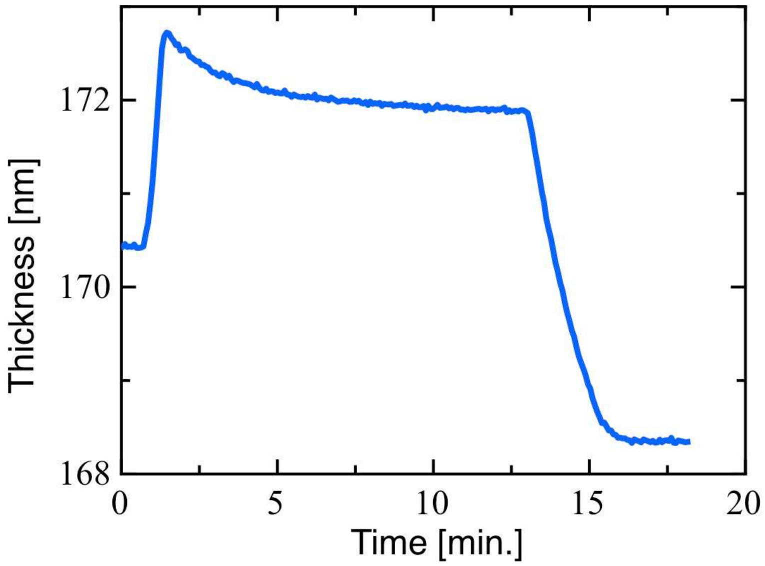

Supplementary Figure 1. Thickness profile of a film without DIO as a function of time, when annealed at the constant temperature of 100ºC, showing the slight (~ 1%) film contraction, compared to the pre-annealed thickness, on cooling from 100ºC to 25ºC.

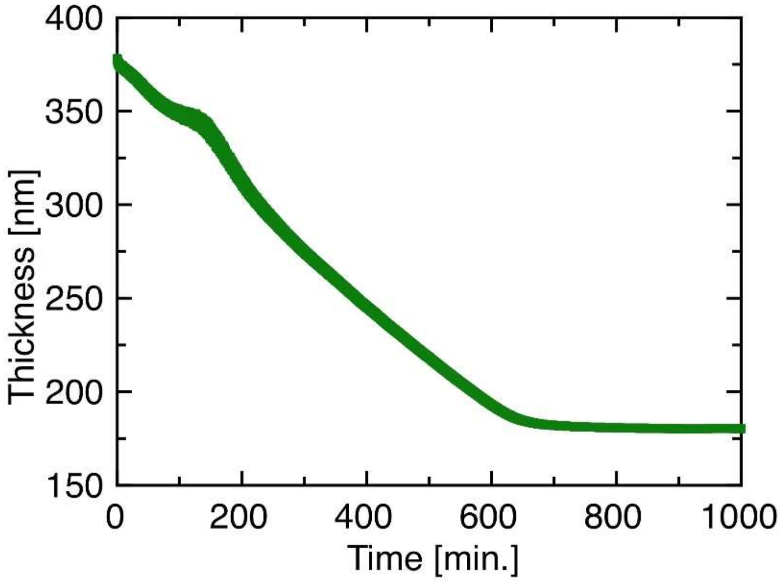

Supplementary Figure 2. Thickness profile of a BHJ film with DIO as a function of time, under room temperature.

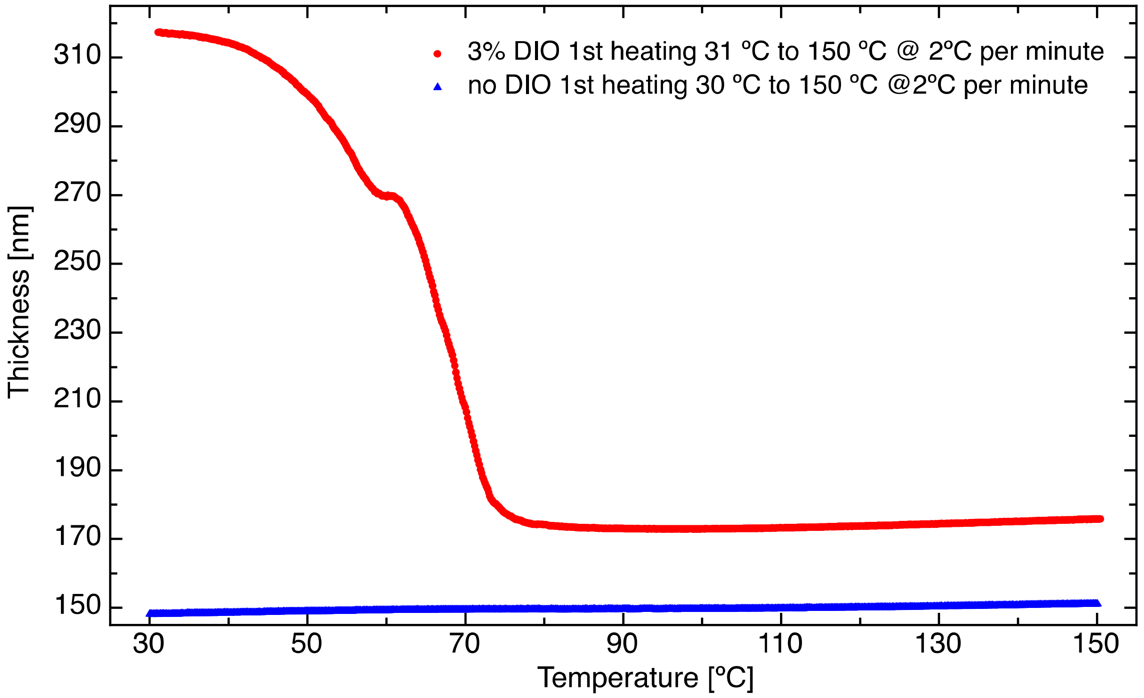

Supplementary Figure 3. Thickness profile of two films (one with DIO and the other without DIO) as a function of temperature, from 30°C to 150°C, corresponding to the first heating scan at a heating rate of 2°C per minute.

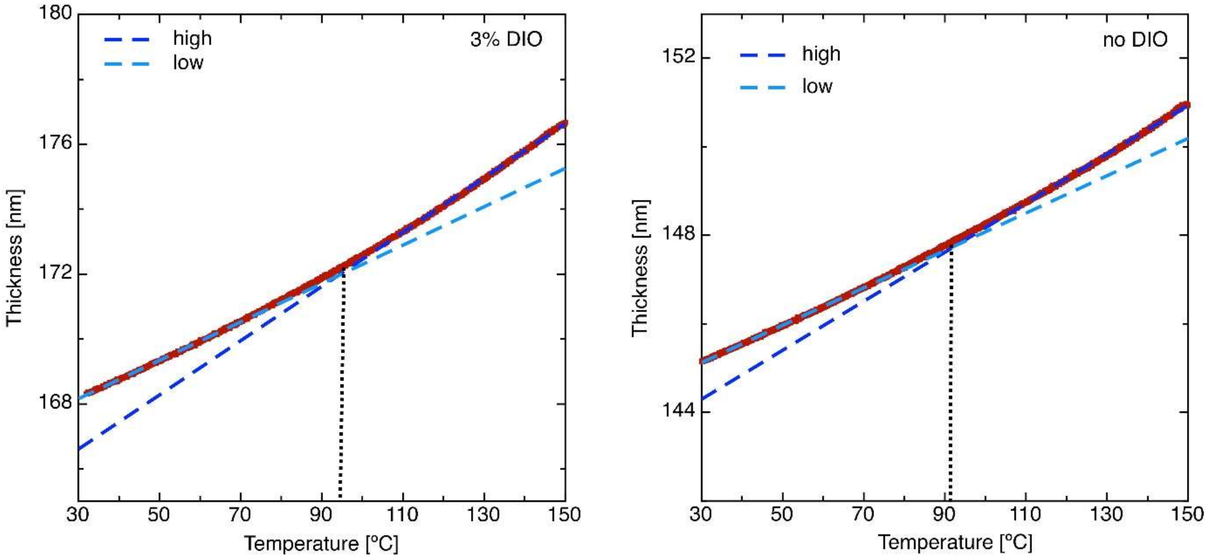

Supplementary Figure 4. Determination of the Tg of the BHJ film as the intercept of the thermal expansion lines above and below Tg as measured on cooling. It must be noted that when measuring these lines, DIO had been already completely removed from the film (with DIO) through evaporation.

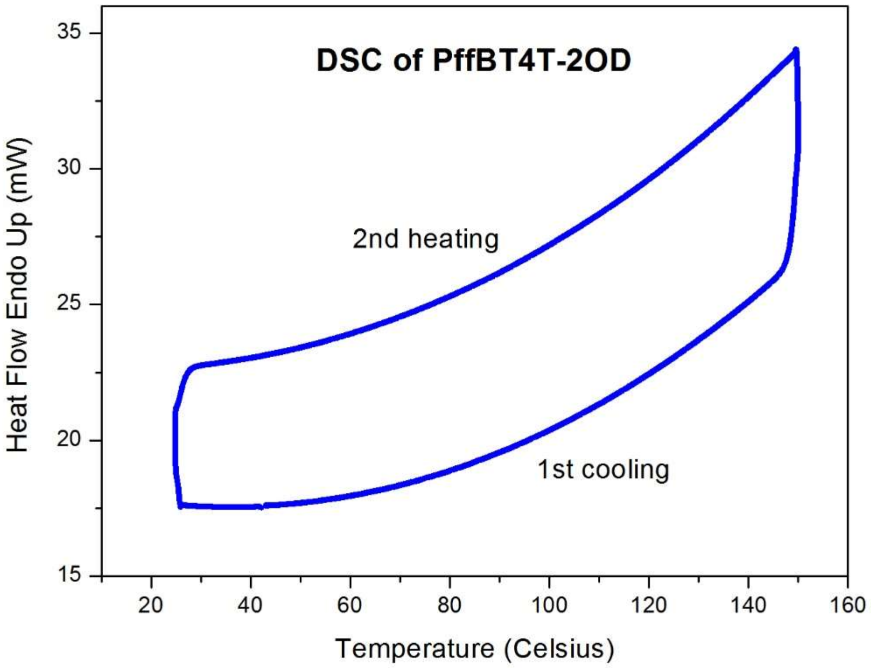

Supplementary Figure 5. Differential scanning calorimetry (scan at a heating/cooling rate of +/-10 ºC.min^{-1}) of the pure PffBT4T-2OD polymer showing the absence of any crystallization peaks in the temperature range 25 – 150 ºC.

(a)

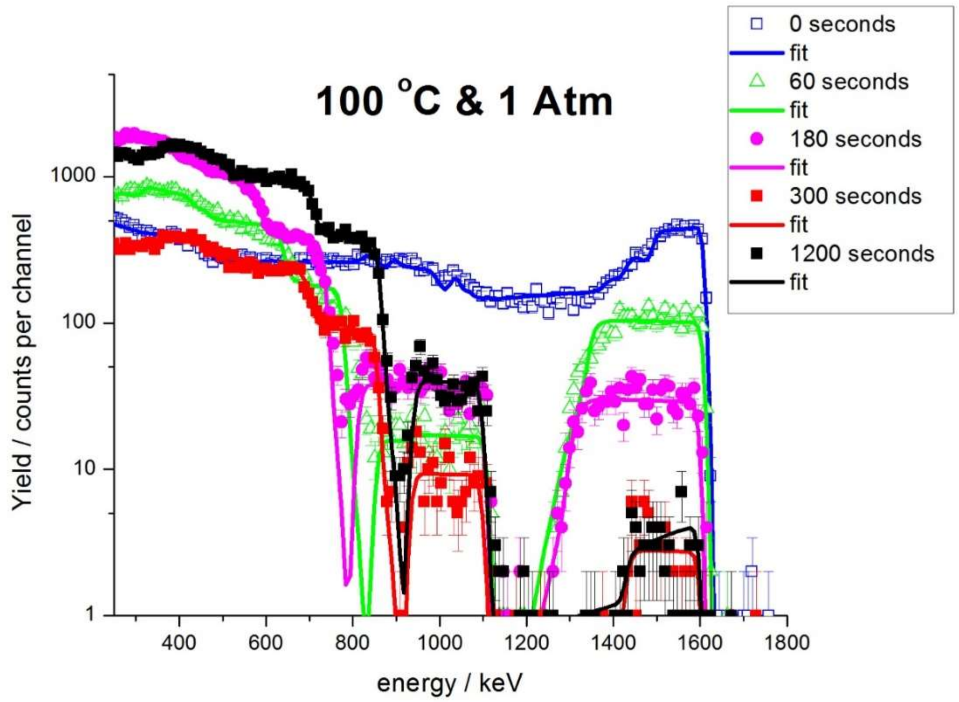
100 °C & 1 Atm
0 seconds
fit
60 seconds
fit
180 seconds
fit
300 seconds
fit
1200 seconds
fit
Yield / counts per channel
1000
100
10
1
400
600
800
1000
1200
1400
1600
1800
energy / keV

(b)

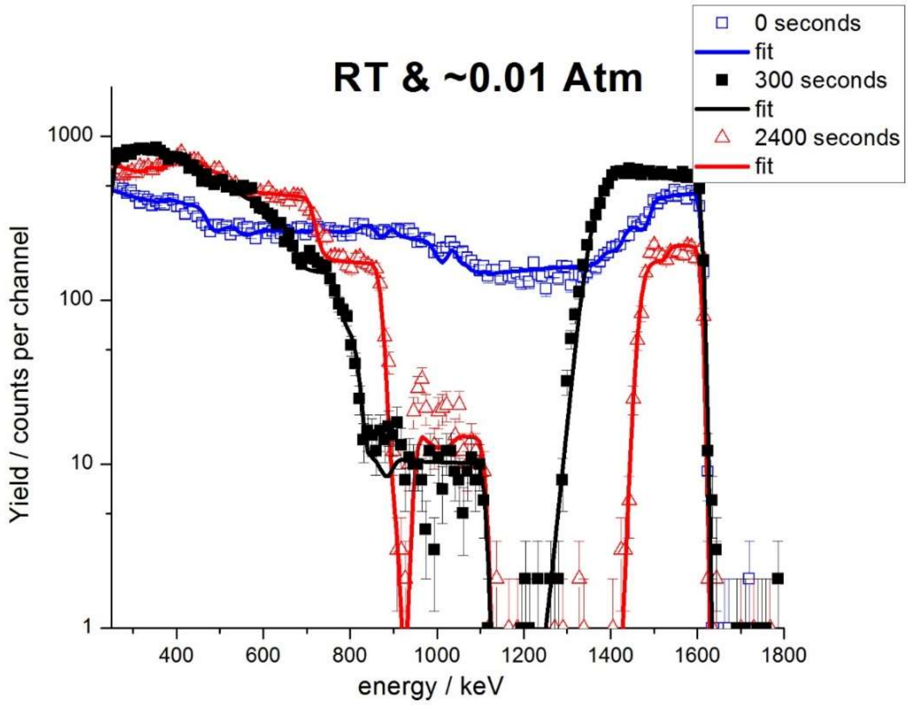
RT & ~0.01 Atm
0 seconds
fit
300 seconds
fit
2400 seconds
fit
Yield / counts per channel
1000
100
10
1
400
600
800
1000
1200
1400
1600
1800
energy / keV

(c)

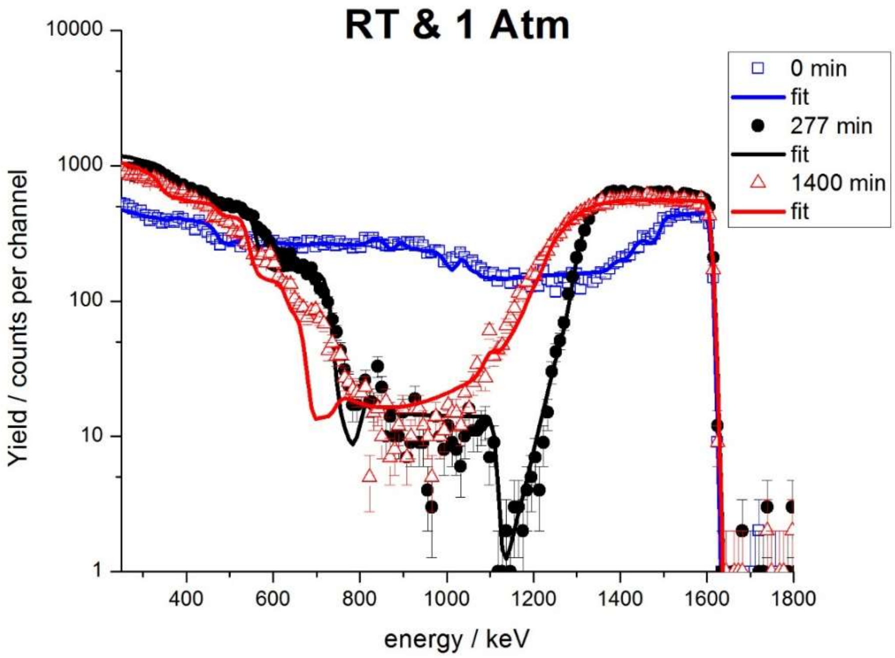

Supplementary Figure 6. Ion-beam results: (a) annealed at 100ºC; (b) under vacuum at RT; (c) ambient conditions.

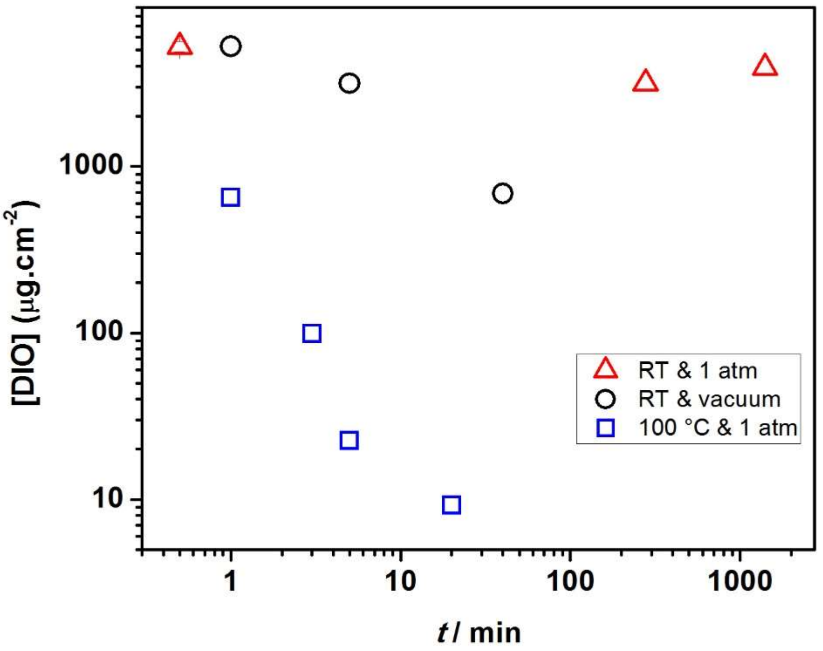

Supplementary Figure 7. Ion-beam results. Concentration of DIO (μg.cm^{-2}) versus time.

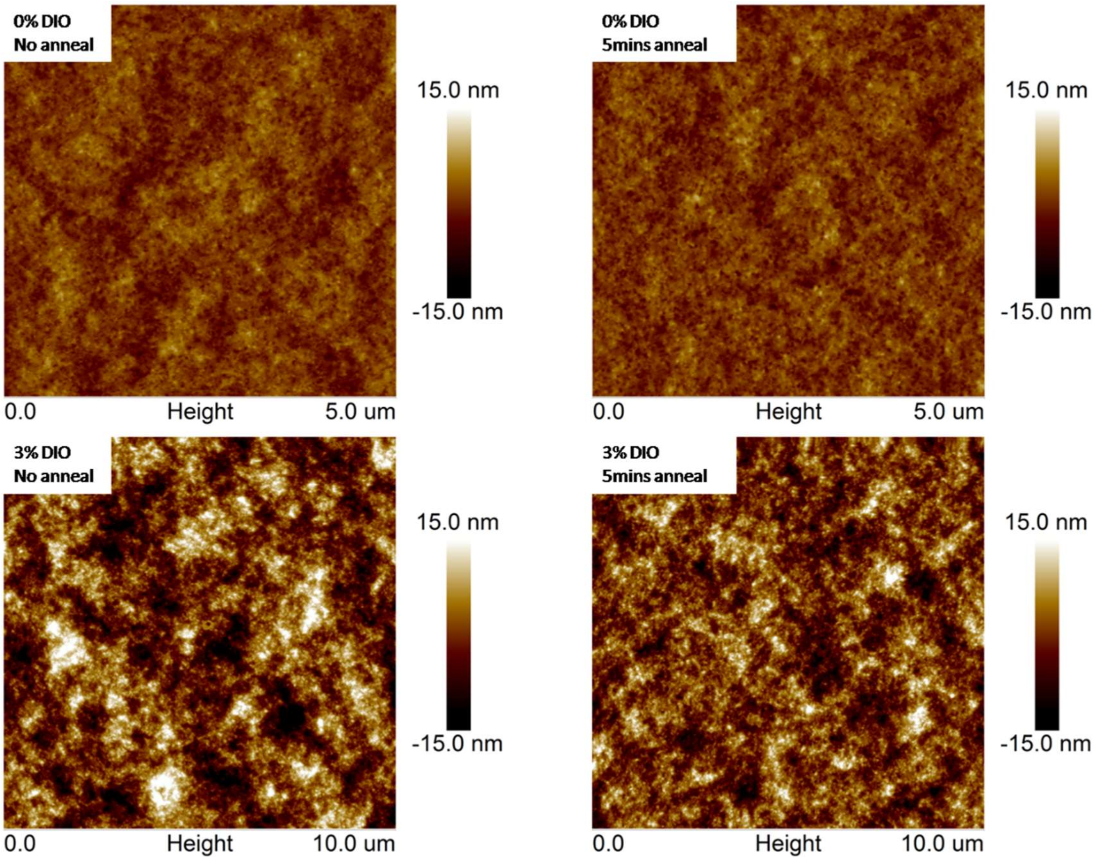

Supplementary Figure 8. SFM images of films with/without different annealing time at 100°C. (a) top morphology images; (b) phase images.

SANS – Mass Fractal model

The Mass Fractal (MF) model defines the scattering in terms of fractal aggregates consisting of spherical primary particles of radius R, such that:

$$\frac{d\Sigma}{d\Omega}(q) = c_{MF}\left[\frac{3[\sin qR - qR\cos(qR)]}{(qR)^3}\right]^2$$

$$\times \frac{\Gamma(D_m-1)\zeta^{(D_m-1)}}{[1+(q\zeta)^2]^{\frac{(D_m-1)}{2}}} \cdot \frac{\sin[(D_m-1)\,tan^{-1}(q\zeta)]}{q} + b$$

where D_m is the mass fractal dimension, ζ is the fractal cut-off length, and c_{MF} is a scale factor that is also proportional to $(\Delta\rho)^2\emptyset_1\emptyset_2$.

In Figure Suppl. Figure 7 is shown the fitting of the experimental data using the MF model. The corresponding fitting parameters are shown in Suppl. Table 1.

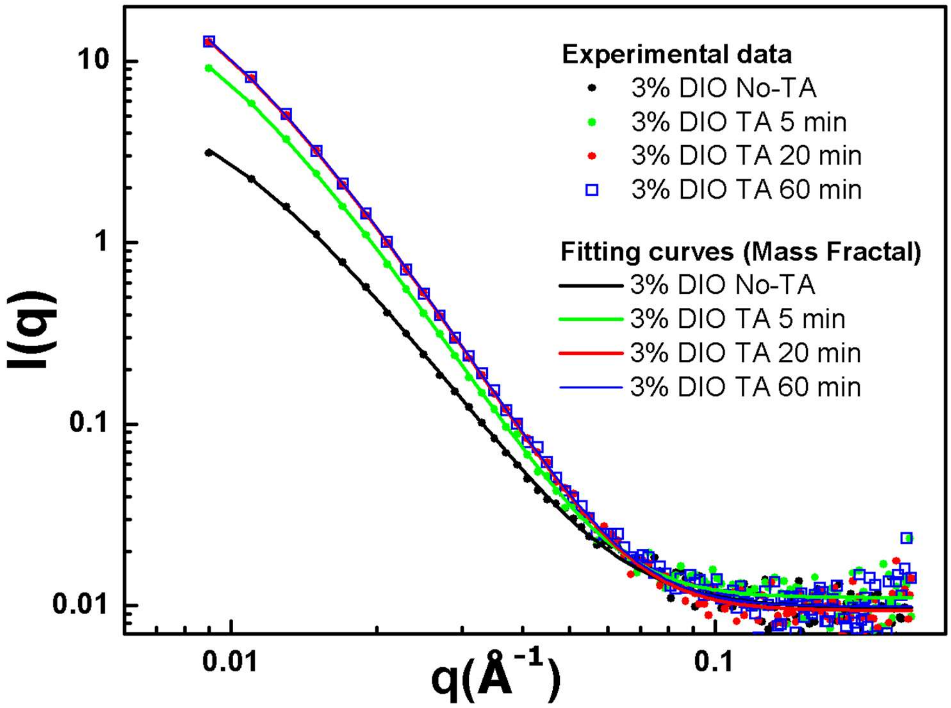

Supplementary Figure 9. SANS intensity data (I(q)) as a function of scattering vector (q) for the non-annealed film and the film annealed for 5, 20 and 60 minutes (symbols). The continuous lines represent fits to the MF model (see above).

	Sample	Mass Fractal Dimension D_m	Scale factor C_{MF}	Characteristic Length ζ (Å)	χ^2/Npts
With DIO 3 wt%	No-annealed	3.00	0.589 x 10^{-5}	91.2 ± 2.1	0.74
	5 min anneal	3.06 (3.03)	0.986 x 10^{-5} (0.129 x 10^{-5})	115.6 ± 2.0 (111.6 ± 9.7)	0.93 (0.73)
	20 min anneal	3.05	1.34 x 10^{-5}	122.0 ± 1.3	1.87
	60 min anneal	3.06	1.32 x 10^{-5}	120.4 ± 1.7	1.18
Without DIO	No-annealed	3.03	0.872 x 10^{-5}	94.5 ± 3.1	0.94
	5 min anneal	3.04	0.871 x 10^{-5}	92.9 ± 3.0	1.35

Supplementary Table 1. Mass fractal dimensions, pre-factors, characteristic lengths (L) and normalized χ^2 values obtained by fitting the experimental data using the Mass Fractal model in the q range 0.009 – 0.1. The values inside the brackets for the DIO sample with 5 min thermal annealing correspond to a sample repeat prepared under similar conditions.

Two-phase random (nonparticulate) system

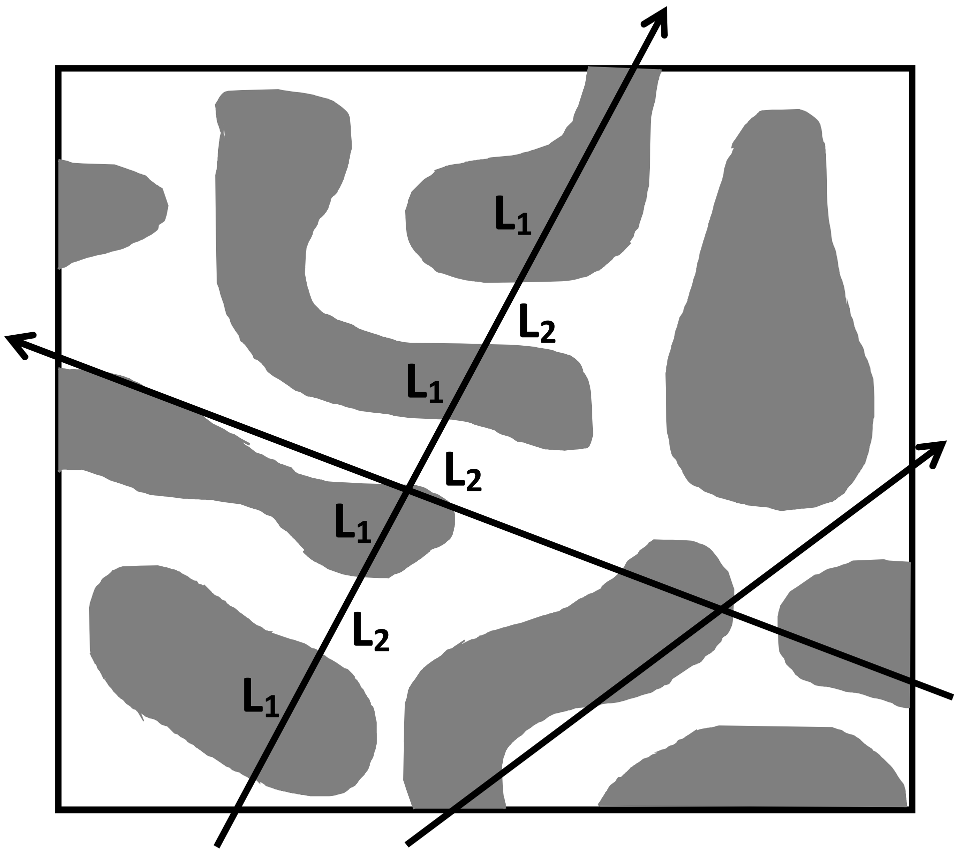

Supplementary Figure 10. Two-phase random (nonparticulate) system. Lines crossing the system in arbitrary directions cut out chords of alternating length L_1 and L_2 between the boundaries of the phases.

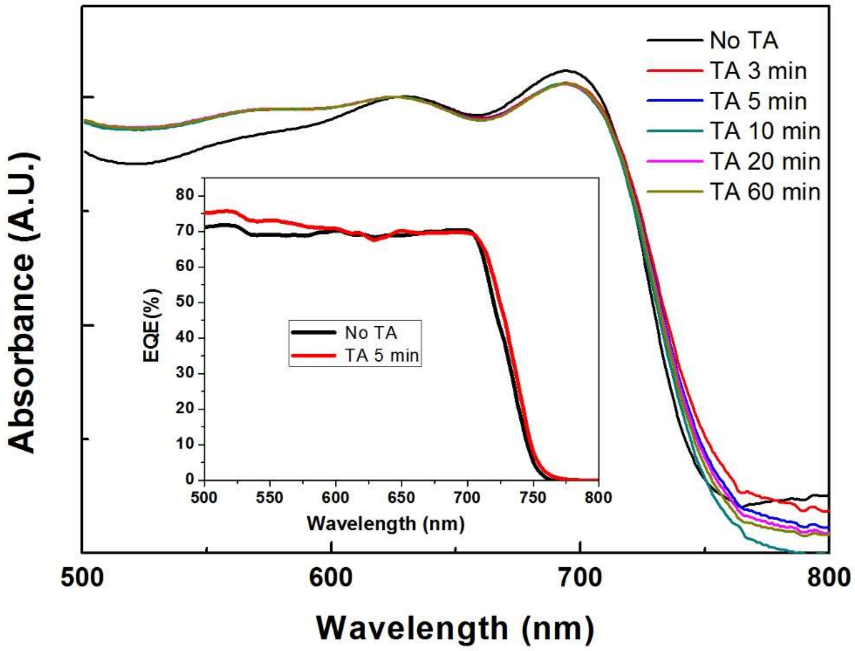

Supplementary Figure 11. UV-Vis absorption of PffBT4T-2OD:$PC_{71}BM$ blend films before and after annealing for various time. All spectra are normalized based on the intensity of their 0-1 transition peak at ~640 nm. The inset shows the EQE results for no-TA and TA 5 min.

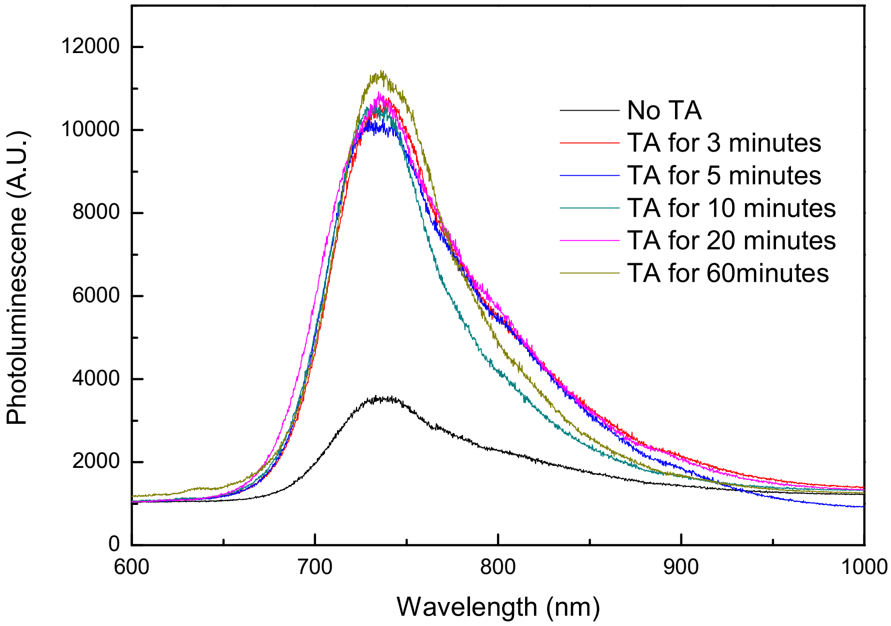

Supplementary Figure 12. Photoluminescence spectra of PffBT4T-2OD:$PC_{71}BM$ blend films before and after annealing for various times.

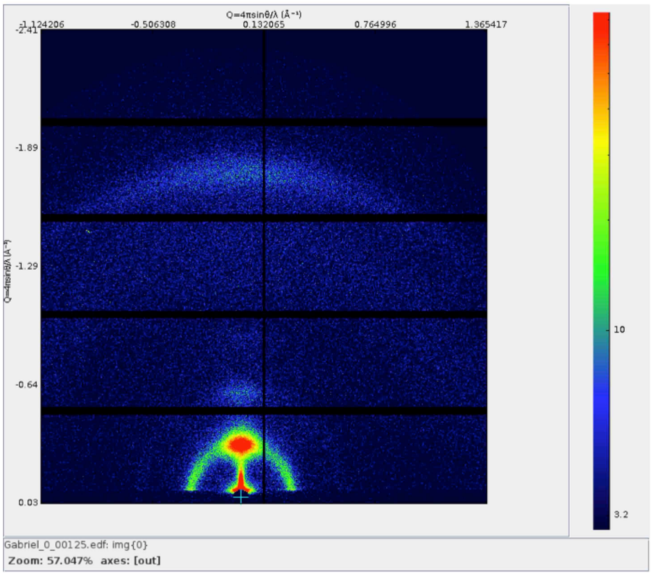

0% DIO, 0 min. thermal anneal

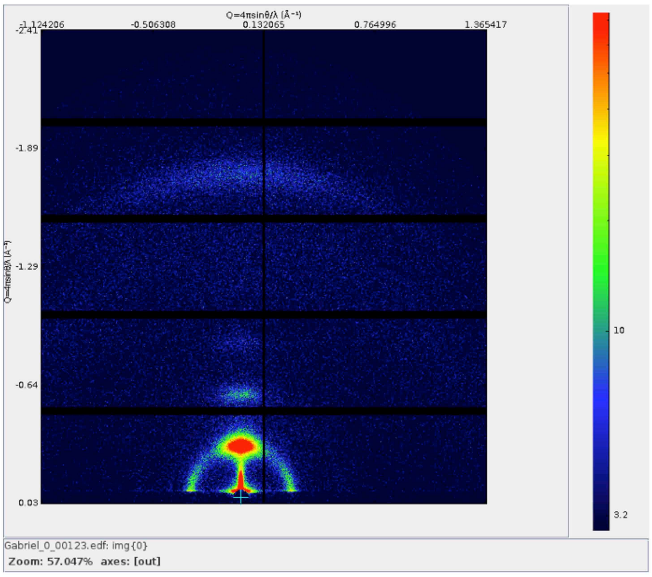

0% DIO, 5 min. thermal anneal

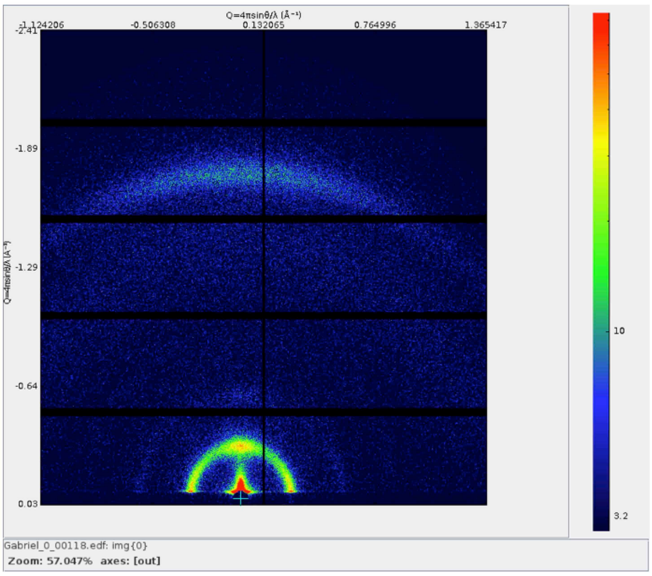

3% DIO, 5 min. thermal anneal

Supplementary Figure 13. Two dimensional GIWAXS detector images for PffBT4T-2OD:$PC_{71}BM$ blend films processed at different processing conditions.

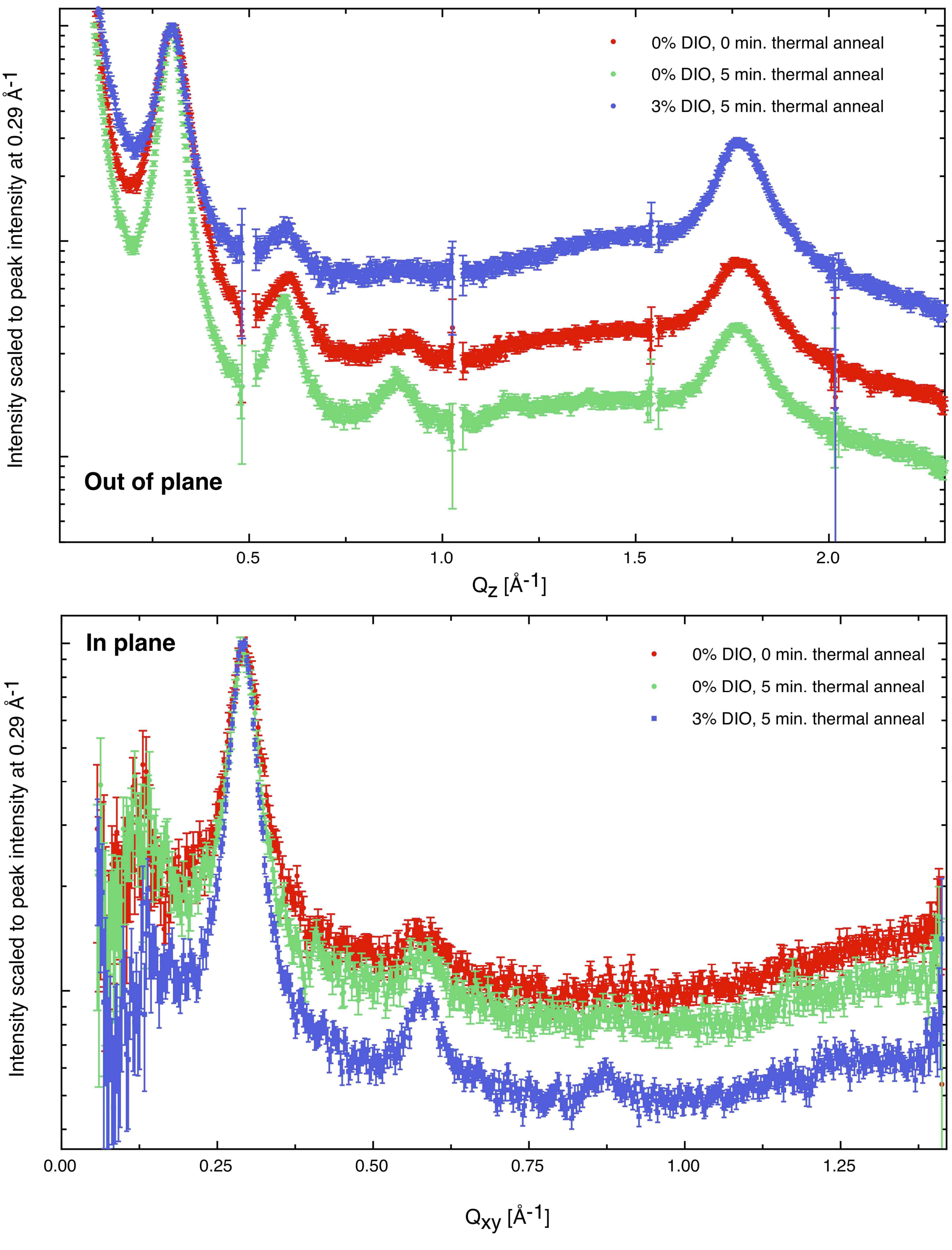

Supplementary Figure 14. 1D GIWAXS. The two dimensional GIWAXS data images were converted to one dimensional data. The image was then integrated (radial integration) using the circle gathering function in Foxtrot (Soleil synchrotron). Two different regions of the 2d detector image were studied to compare the in plane and out of plane ordering.
